# Pathway from Earthquake Fear to Post-Traumatic Growth: The Roles of Resilience, Self-Efficacy, and Positive Childhood Memories Among Survivors of the 2023 Türkiye Earthquakes

**DOI:** 10.1007/s11126-025-10113-1

**Published:** 2025-01-23

**Authors:** Yakup İme, Rumeysa Hoşoğlu Kama, Nihan Çitemel Arslan

**Affiliations:** 1 Necmettin Erbakan University, Toros Mah, Üniversite Cad., No:10, Ereğli, Konya, Ereğli Turkey; 2 Necmettin Erbakan University, Toros Mah. Universite Cad., Ereğli, Konya, 42310 Turkey

**Keywords:** Earthquake fear, Resilience, Self-efficacy, Positive childhood memories, Post-traumatic growth

## Abstract

Posttraumatic growth is essential for understanding how individuals process trauma and adapt psychologically in the aftermath of seismic events. This study aims to explore the mediating effects of resilience, self-efficacy, and positive childhood memories on the relationship between fear of earthquakes and post-traumatic growth among survivors of the 2023 Türkiye earthquake (*N* = 423). The results of a multi-mediation analysis indicated that earthquake fear indirectly influenced post-traumatic growth through resilience, self-efficacy, and positive childhood memories. Consequently, these factors may serve as protective mechanisms promoting post-traumatic growth in earthquake survivors. Implementing strategies to enhance resilience and self-efficacy, as well as fostering positive childhood memories, may be essential for mitigating the adverse effects associated with earthquakes.

## Introduction

The 2023 earthquakes in Türkiye have highlighted growing concerns about the psychological toll on survivors. Fear of earthquakes, a typical reaction to such traumatic experiences, can lead to various mental health challenges, including posttraumatic stress disorder (PTSD) and depression [36]. Earthquake fear is a prevalent emotional response experienced by individuals during and after seismic events. This fear can significantly influence psychological outcomes, including the development of post-traumatic stress disorder (PTSD) and other anxiety-related disorders. Research indicates that the intensity of fear felt during an earthquake is a strong predictor of PTSD symptoms in survivors, as it is closely related to the perceived threat to life and property [35, 42]. Moreover, the persistence of reminders of traumatic events, such as damaged buildings and ongoing aftershocks, can exacerbate feelings of fear and anxiety, leading to prolonged psychological distress [35]. Although the aftermath of an earthquake often brings intense fear and psychological challenges, studies indicate that this traumatic experience can also catalyze post-traumatic growth, leading to a deeper sense of purpose and emotional strength [40].

Understanding the relationship between earthquake fear and posttraumatic growth (PTG) is crucial, as it can help guide strategies aimed at strengthening resilience and fostering positive mental health outcomes for those affected. Post-traumatic growth (PTG) refers to the positive psychological changes that individuals may experience following a traumatic event, such as an earthquake [44]. PTG in earthquake survivors, as with individuals experiencing trauma from various other situations, is rooted in the ability to find meaning and personal transformation after profound suffering [2]. Theoretical foundations of PTG suggest that such growth often emerges from the cognitive and emotional processes triggered by trauma [37]. In the context of earthquakes, survivors often report significant transformations in their personal relationships, self-perception, and life philosophies because of their experiences [20]. The phenomenon of post-traumatic growth highlights the complex interplay between trauma and recovery, suggesting that even in the face of devastating events, individuals can emerge with newfound strength and insight. Research indicates that the trauma associated with earthquakes can serve as a catalyst for personal growth, leading individuals to develop a greater appreciation for life and a deeper understanding of their own strengths [2]. This resilience not only aids in recovery but also contributes to the likelihood of experiencing PTG, as those who are better equipped to handle stress are often more open to finding meaning in their suffering [18]. According to İkizer and Özel [45], recovery following trauma is both an individual capacity and a function of the individual’s context and culture. They suggest that searching for cultural explanations and investigating whether spiritual tendencies would mediate the relationships between resilience and growth may be important for future research. Furthermore, the struggle to cope with the aftermath of an earthquake can lead to increased resilience, as individuals learn to navigate their fears and uncertainties [15].

Resilience, the ability to adapt and recover from hardship, plays a key role in influencing psychological outcomes following trauma [33]. Research suggests that resilience can significantly shape how individuals cope with traumatic events, including natural disasters. For instance, studies have shown that resilience can act as a buffer between trauma exposure and PTSD, with more resilient individuals being better equipped to manage their emotional reactions to trauma [21, 23]. Additionally, resilience interacts with early life experiences, where negative childhood events can influence an individual’s capacity to cope with later trauma, affecting their psychological responses [11, 23]. This suggests that resilience not only helps reduce the negative impact of trauma but also facilitates posttraumatic growth, allowing individuals to find meaning and personal development in the wake of distressing events.

Beyond resilience, factors such as self-efficacy and positive childhood memories are also important in understanding how earthquake fear can influence posttraumatic growth. Self-efficacy, or the belief in one’s ability to control life events, has been linked to more effective coping strategies and lower PTSD levels [8, 22]. This is particularly relevant in the context of earthquakes, where the unpredictability and destruction can lead to significant psychological distress; those who believe in their capacity to cope are more likely to engage in proactive recovery behaviors [6]. Individuals with high self-efficacy are more likely to engage in proactive coping behaviors, which can enhance resilience and lead to better psychological outcomes after trauma. Similarly, positive childhood memories can act as a protective factor, helping individuals maintain a sense of stability and hope during times of crisis, further promoting resilience [22, 41]. Childhood memories, particularly those of trauma or adversity, can act as foundational experiences that shape an individual’s ability to process and make sense of later life challenges. Early childhood experiences—such as neglect, abuse, or loss—can either impede or facilitate the process of PTG, depending on the individual’s coping mechanisms and the meaning they assign to those events [43]. Positive childhood memories often serve as a foundation for emotional resilience and psychological well-being throughout life. These memories can encompass a range of experiences, from joyful family gatherings to simple moments of play, which contribute to a child’s sense of security and belonging. Research indicates that such positive experiences can significantly influence an individual’s coping mechanisms and overall mental health in later years [29]. While positive childhood memories are vital for healthy development, the interplay between these memories and experiences of trauma can shape an individual’s capacity for growth and adaptation in the face of future challenges [28].Integrating these factors into the study of posttraumatic growth is essential, as they collectively contribute to an individual’s ability to navigate the emotional challenges of trauma and facilitate recovery.

### Present Study

The aftermath of significant seismic events often leaves survivors grappling with a myriad of psychological challenges, most notably heightened levels of fear associated with future earthquakes. This fear can profoundly impact an individual’s mental health and overall well-being, potentially leading to maladaptive coping strategies and prolonged psychological distress [10]. However, not all survivors experience negative outcomes; some demonstrate remarkable resilience and experience PTG, characterized by personal development following adversity [38]. Understanding the interplay between earthquake-related fear and PTG is crucial for developing effective psychological interventions and support mechanisms for survivors. By exploring how fear influences PTG, we can gain insights into the psychological processes that facilitate recovery and adaptation in the face of natural disasters.

This study aims to investigate the mediating roles of resilience, self-efficacy, and positive childhood memories in the relationship between earthquake fear and posttraumatic growth. These factors are hypothesized to serve as protective mechanisms that enhance an individual’s capacity to cope with trauma and foster personal growth. Resilience may provide the emotional strength to navigate the challenges posed by fear, while self-efficacy can empower individuals to take proactive steps toward recovery. Positive childhood memories might serve as a source of comfort and stability, further buffering against the detrimental effects of trauma. The findings of this research could contribute significantly to the fields of psychology and disaster management by informing therapeutic practices and interventions aimed at enhancing PTG in earthquake survivors, ultimately leading to improved mental health outcomes and community resilience following seismic events. This study seeks to explore the mediating roles of resilience, self-efficacy, and positive childhood memories in the relationship between earthquake fear, and post-traumatic growth among survivors of the 2023 Türkiye earthquakes. Drawing on existing literature, the study posits two hypotheses. First, it is expected that earthquake fear will exhibit a positive correlation with post-traumatic growth. Second, it is hypothesized that the relationship between earthquake fear and post-traumatic growth will be mediated by resilience, self-efficacy, positive childhood memories.

## Method

### Participants

The study was conducted roughly 10 months after the Türkiye Earthquake that struck on February 6, 2023. The requirement for participation in the study was being affected by the earthquakes in Türkiye on February 6, 2023. Participants were selected through convenience sampling. Participants were informed about the purpose and duration of the study before participating in the study and were told that they could leave the study at any time. The participants, all survivors of the earthquake, were living in various situations such as state-run dormitories, with close family members in different provinces, or in buildings that had not been damaged by the earthquake in the affected region. All individuals included in the study resided in cities directly affected by the earthquake. Data was gathered through face-to-face interviews with the participants. In total, 423 earthquake survivors took part in the research, including 154 males (36.4%) and 269 females (63.6%). In terms of educational background, 24 participants (5.7%) had completed primary school, 268 (63.4%) had completed high school, 115 (27.2%) had completed undergraduate degree, and 16 (3.8%) held a master’s degree or higher. Regarding marital status, 336 participants (79.4%) were single, 79 (18.7%) were married, and 8 (1.9%) were divorced. Additionally, 29 participants (6.9%) reported having received psychiatric treatment, while 394 participants (93.1%) indicated they had not. The participants’ ages ranged from 18 to 40, with an average age of 27.87 (*SD* = 6.36).

### Instruments

#### Earthquake Fear Scale

The scale was developed by Satıcı et al. [30]. The scale, which consists of seven items, is one-dimensional and is a 5-point Likert-type scale. The scale’s scoring ranges from 1 to 5 (1-strongly disagree, 5-strongly agree). The lowest score that can be obtained from the scale is 7, and the highest score is 35. A high score obtained from the scale indicates a high fear of earthquakes. The Cronbach’s alpha value of the scale was calculated as 0.91.

#### Brief Resilience Scale

The scale, developed by Smith et al. [32], was adapted into Turkish by Doğan [14]. The scale consists of a single dimension and contains a total of 6 items. Each item is scored between 1 (not at all appropriate) and 5 (completely appropriate). The lowest score that can be obtained from the scale is 6, and the highest score is 30. A high score on the scale indicates high resilience. The internal consistency coefficient of the scale was calculated as 0.83.

#### General Self-Efficacy (GSE) Scale

The scale developed by Schwarzer and Jerusalem [31], was adapted to Turkish by Aypay [5]. The scale consists of two dimensions and contains a total of ten items. Each item is scored between 1 (completely false) and 4 (completely true). The lowest score that can be obtained from the scale is 4, and the highest score is 40. A high score from the scale indicates high general self-efficacy. Cronbach alpha internal consistency coefficients were calculated as 0.91.

#### Early Memories of Warmth and Safeness Scale

The scale, developed by Richter et al. [26], was adapted to Turkish by Akın et al. [1]. The scale consists of a single dimension and includes a total of twenty items. Each item is scored between 1 (never) and 5 (always). The lowest score that can be obtained from the scale is 20, and the highest score is 100. A high score obtained from the scale indicates the abundance of happy and peaceful childhood memories. The internal consistency coefficient of the scale was calculated as 0.94.

#### Post-Traumatic Growth Inventory

The Post-Traumatic Growth Scale, developed by Tedeschi and Calhoun (1996), was adapted into Turkish by Aydın and Kabukçuoğlu [4]. The scale consists of five dimensions and a total of twenty-one items. Each item is scored between 0 and 5. The lowest score that can be obtained from the scale is 0, and the highest score is 105. A high score indicates that the person experienced a high level of growth after the traumatic experience. The internal consistency coefficient of the scale was calculated as 0.93.

#### Data Analysis

Initially, statistical assumptions, descriptive statistics, and correlations among the study variables were assessed, with a significance level established at 0.05. The normality of the data was evaluated by computing skewness and kurtosis values [34]. Pearson correlation analysis was employed to examine the relationships between the study variables. Subsequently, mediation analysis was performed using the Process macro for SPSS. The Hayes Process Macro offers a user-friendly, flexible, and statistically robust method for examining complex models involving mediation, moderation, and conditional processes, making it a preferred choice for many researchers over other alternatives [16]. This method facilitates the calculation of both direct and indirect relationships between variables through one or more mediators [16]. The mediation model was determined by calculating the standard path coefficient values (β) among the variables. To assess the significance of the indirect effects, 5,000 bootstrap samples were utilized (95% CI). An indirect effect is deemed significant if the bootstrap confidence interval does not include zero [16]. All analyses were conducted using SPSS version 28.

## Results

As seen in Table 1, earthquake fear was significantly negatively correlated with resilience scores. Positive correlations were found between earthquake fear, positive childhood memories and post-traumatic growth. Significant positive correlations were found between resilience, self-efficacy, and post-traumatic growth. Also, significant positive correlations were found between self-efficacy, positive childhood memories and post-traumatic growth. Lastly, significant positive correlation was found between positive childhood memories and post-traumatic growth. Moreover, the values of skewness and kurtosis were determined to be within acceptable limits, indicating that the data followed a normal distribution [34].

**Table 1:** Correlations and descriptive statistics (*N* = 423)

Variables	1	2	3	4	5
1. Earthquake fear	1				
2.Resilience	− 0.36^**^	1			
3. Self-efficacy	− 0.08^**^	0.49^**^	1		
4.Positive childhood memories	0.14^**^	0.07	0.31**	1	
5.Post traumatic growth	0.17^**^	0.27^**^	0.54^**^	0.31^**^	1
*M*	3.15	2.95	3.00	3.66	3.19
*SD*	1.02	0.76	0.55	0.97	0.79
Skewness	− 0.053	− 0.311	− 0.027	− 0.613	− 0.444
Kurtosis	− 0.624	0.614	− 0.331	− 0.085	1.18

Note. **p <.001

### Mediational Analyses

**Fig. 1:**
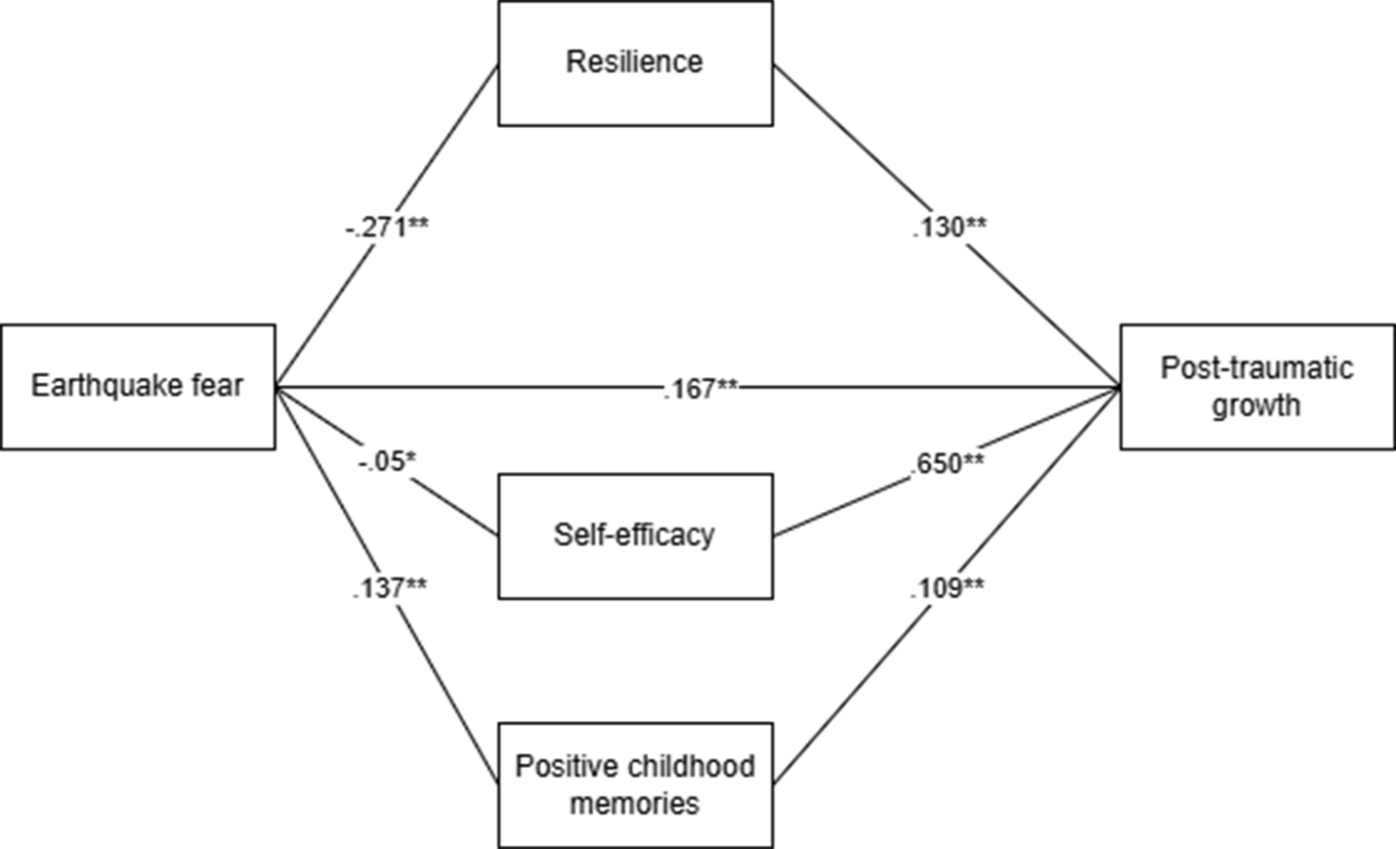
Parallel multiple mediation role of resilience, self-efficacy, and positive childhood memories in the relation between earthquake fear and post-traumatic growth. **p <.001, *p<. 05

The findings regarding the role of parallel mediating variables in the relationship between earthquake fear and post traumatic growth are given in Fig. 1.

The direct effect of earthquake fear (*β* = 0.167, *p* <.001) on post traumatic growth was statistically and positively significant. The findings further indicate that all simple path mediation coefficients are also statistically significant (*p* <.001). Primarily, the findings show that earthquake fear has a direct statistically significant negative effect on resilience (*β* = −0.271, *p* <.001), self-efficacy (*β* = −0.05, *p* <.05), and positive child memories (*β* = 0.137, *p* <.001). Finally, the effects of resilience (*β* = 0.130, *p* <.001), self-efficacy (*β* = 0.650, *p* <.001), and positive childhood memories (*β* = 0.109, *p* <.001) on post traumatic growth were also found to be positively significant.

Displays the results concerning the indirect effects of the three mediating variables, with the standardized Beta coefficients provided along with their 95% confidence intervals (CI). Earthquake fear exerts an indirect effect on post-traumatic growth via resilience (= --0.035, SE = 0.014, *p* <.05), self-efficacy (= − 0.013, SE = 0.019, *p* <.05), and positive childhood memories ( = 0.015, SE = 0.007, *p* <.001)table 2.

**Table 2:** Indirect effect of earthquake fear on post-traumatic growth via resilience, self-efficacy, positive childhood memories

Path	Indirect effects	*SE*	Boot LLCI	Boot ULCI
Earthquake fear→ resilience→ post-traumatic growth	− 0.035	0.014	− 0.063	− 0.007
Earthquake fear→ self-efficacy→ post-traumatic growth	− 0.013	0.019	− 0.051	− 0.005
Earthquake fear→ positive childhood memories→ post-traumatic growth	0.015	0.007	0.002	0.032

## Discussion

The objective of this study was to examine the mediating role of resilience, self-efficacy, and positive childhood memories in the relationship between earthquake fear, and post-traumatic growth among survivors of the 2023 Türkiye earthquakes. The findings reveal a positive correlation between earthquake fear and post-traumatic growth. Furthermore, the results illustrate the indirect effects of earthquake fear on post-traumatic growth through the mediators of resilience, self-efficacy, and positive childhood memories. These insights suggest that resilience, self-efficacy, and post-traumatic growth may act as protective factors that enhance post-traumatic growth among earthquake survivors.

The finding that there is a positive relationship between earthquake-related fear and PTG adds a nuanced perspective to the understanding of how individuals respond to trauma. Previous research has shown mixed results regarding the link between anxiety and PTG. On one hand, some studies support this positive relationship, suggesting that individuals who experience higher levels of anxiety or distress following a traumatic event may be more likely to engage in deep self-reflection, reevaluate their life priorities, and experience positive psychological changes over time [37]. For instance, research on natural disaster survivors, such as those affected by earthquakes or hurricanes, has highlighted that the emotional turmoil and fear associated with the traumatic event can catalyze personal growth, such as increased resilience, a greater sense of purpose, and enhanced relationships [10]. However, other studies suggest that anxiety may impede post-traumatic growth or that the relationship between these variables is not as straightforward. Some research indicates that while trauma survivors may initially experience high levels of distress and anxiety, this does not always translate into growth. For example, a study by Nolen-Hoeksema [24] found that excessive anxiety or rumination could hinder psychological recovery and prevent individuals from making the adaptive changes needed for PTG. Furthermore, fear may result in long-term avoidance behaviors or avoidance coping strategies, which could negatively affect the process of growth and healing. Similarly, some studies suggest that individuals who experience prolonged distress may become stuck in a state of emotional dysregulation, making it harder for them to perceive the benefits or transformative aspects of their trauma [13]. Additionally, the nature of the trauma itself and the resources available to survivors can influence whether anxiety fosters or inhibits PTG [25]. Therefore, while fear and anxiety may initially seem to promote personal reflection, the trajectory toward PTG is likely influenced by a complex interplay of individual and contextual factors. In sum, while this study’s finding that earthquake fear and PTG are positively correlated adds valuable insight into the mechanisms of growth after trauma, it is important to recognize the diversity of responses to traumatic events, with some survivors facing significant barriers to growth despite experiencing high levels of anxiety.

The finding that resilience, self-efficacy, and positive childhood memories partially mediate the relationship between earthquake fear and post-traumatic growth provides important insights into the psychological mechanisms that underlie recovery after trauma. Resilience, defined as the ability to adapt to and recover from adversity, has been well-established as a crucial factor in promoting PTG [9]. In the context of earthquake survivors, resilience likely enables individuals to cope with the intense fear and distress that accompany natural disasters, while facilitating the positive psychological transformations that characterize PTG [39]. The mediation effect of resilience in the present study suggests that individuals with higher levels of resilience may be better equipped to transform fear and anxiety into growth experiences, rather than being overwhelmed by them.

Similarly, self-efficacy been shown to play a significant role in how individuals respond to traumatic events. Bandura’s [7] theory of self-efficacy posits that those who perceive themselves as capable of handling stressors are more likely to engage in adaptive coping strategies and experience greater psychological well-being. In the case of earthquake survivors, high self-efficacy could enable individuals to take proactive steps toward rebuilding their lives after the disaster, fostering a sense of personal growth [17]. Individuals with higher self-efficacy tend to perceive traumatic events, like earthquakes, as challenges they can handle, rather than insurmountable obstacles. This mindset fosters resilience, encouraging survivors to take proactive steps toward recovery and personal development [3]. This aligns with studies showing that self-efficacy is positively related to both post-traumatic recovery and PTG [19]. The present study’s finding that self-efficacy mediates the relationship between earthquake fear and PTG underscores the importance of promoting self-confidence and a sense of control in disaster recovery efforts. Therefore, self-efficacy is a key factor that can facilitate the process of posttraumatic growth, helping survivors rebuild and emerge stronger from the experience.

Positive childhood memories, as another mediator, may also play a pivotal role in how individuals process and recover from trauma. Research has suggested that individuals with positive early life experiences are better equipped to cope with later-life adversities [27]. These memories can provide a psychological foundation of safety and stability that helps individuals navigate the emotional chaos of traumatic events. In the context of PTG, positive childhood memories may serve as a reservoir of resilience, offering individuals the psychological resources to reinterpret the trauma in a way that leads to growth, rather than despair [46]. The study’s findings suggest that these memories may buffer the distress caused by earthquake fear, allowing survivors to access a sense of continuity and meaning in their lives, which is a key component of PTG [25]. In the context of earthquake survivors, individuals with positive childhood memories—such as secure attachment to caregivers, stable family environments, and a strong sense of community—may be better equipped to process and adapt to the aftermath of the disaster. These early experiences contribute to healthier coping mechanisms, such as emotional regulation, optimism, and social connectedness, which are essential for managing the stress and psychological impact of post-earthquake trauma [12].

These three mediators—resilience, self-efficacy, and positive childhood memories—do not operate in isolation. Instead, they likely interact in complex ways. For example, resilience may influence the development of self-efficacy, as individuals with greater resilience might be more likely to believe in their ability to overcome challenges. Similarly, positive childhood memories may foster resilience by reinforcing a sense of security and continuity in the face of trauma. Taken together, these factors form a network of psychological resources that help individuals navigate the earthquake fear associated with trauma and transform those emotions into opportunities for growth.

### Limitations

Despite the valuable insights provided by this study on the relationship between earthquake fear and posttraumatic growth (PTG) among survivors of the 2023 Türkiye earthquake, several limitations should be noted. First, the study’s cross-sectional design limits the ability to draw causal inferences about the relationships between the variables. Since data were collected at a single point in time, it is unclear whether earthquake fear leads to posttraumatic growth through the mediating variables of resilience, self-efficacy, and positive childhood memories, or whether the reverse direction of influence is possible. Longitudinal studies would be necessary to establish causality and examine how these relationships unfold over time as individuals continue to process their traumatic experiences. Second, the study relied on self-reported measures, which may be subject to biases such as social desirability, recall bias, or individual differences in the interpretation of the questions. These biases could affect the accuracy of the reported levels of earthquake fear, resilience, self-efficacy, and PTG. Third, the sample was drawn from a specific geographic area in Türkiye, and although the participants were earthquake survivors, the findings may not be generalizable to other populations affected by different types of traumas or to individuals in different cultural contexts. The fact that participants were reached face-to-face might also introduce sampling biases, as those who were more willing or able to participate in the study may differ from those who did not. Additionally, the lack of control for external variables such as social support, financial stability, or pre-existing mental health conditions limits the ability to fully understand the factors contributing to posttraumatic growth. Future research should address these limitations by employing longitudinal designs, using diverse methods of data collection, and considering a broader range of influencing factors.

## Conclusion

In conclusion, the identified positive correlation between earthquake fear and post-traumatic experiences in survivors emphasizes the complex relationship between emotional reactions and psychological outcomes following traumatic events. This finding suggests that fear, while typically viewed as a negative emotion, could also function as a trigger for increased awareness and the development of coping strategies that support post-traumatic growth. Recognizing this connection is essential for designing effective interventions that assist survivors, as it underscores the importance of addressing both the fear of earthquakes and the resulting psychological effects. Future studies should delve deeper into the mechanisms behind this relationship and explore how fear might be harnessed as a tool to enhance resilience and promote adaptive coping in the aftermath of natural disasters. Understanding this relationship is essential for mental health professionals and disaster response teams, as it highlights the need for interventions that address fear management as a precursor to facilitating PTG.

Furthermore, the positive associations found between resilience, self-efficacy, and positive childhood memories with PTG suggest that these factors play a crucial role in mediating the adverse effects of earthquake fear. Resilience equips individuals with the emotional tools necessary to face challenges, while self-efficacy fosters a sense of control and agency, encouraging proactive coping strategies. Positive childhood memories may serve as a source of strength, providing comfort and stability in times of distress. These insights not only contribute to the theoretical understanding of trauma recovery but also have practical implications for developing targeted interventions. By enhancing resilience and self-efficacy, and by fostering positive recollections of childhood, mental health practitioners can better support earthquake survivors in their journeys toward posttraumatic growth. This research advocates for a holistic approach to disaster recovery, emphasizing the importance of psychological well-being in rebuilding lives after trauma.

### Practical Implications

The findings of this research offer several practical implications for mental health professionals, disaster response teams, and community organizations working with earthquake survivors. First and foremost, the relationship between earthquake fear and posttraumatic growth highlights the necessity for targeted interventions aimed at fear management. Programs designed to address and alleviate fear could play a critical role in enabling individuals to engage more fully with their recovery process. Additionally, fostering resilience should be a key focus in therapeutic practices; interventions that build emotional strength can empower survivors to better navigate the aftermath of trauma. Similarly, enhancing self-efficacy through skills training and coping strategies can instill a sense of agency, encouraging survivors to take proactive steps toward their healing.

Moreover, incorporating discussions of positive childhood memories into therapy can provide a valuable framework for reinforcing identity and stability during the recovery process. Mental health practitioners might consider using narrative therapy techniques to help survivors recall and integrate these positive experiences, thereby bolstering their psychological resources. Community organizations can also play a vital role by providing support groups that focus on shared experiences, which can foster resilience and enhance social connections. Training workshops for first responders and community leaders could further enhance their understanding of these dynamics, equipping them to provide more effective support. Ultimately, a comprehensive approach that addresses fear while promoting resilience, self-efficacy, and positive memories can significantly improve the mental health outcomes of earthquake survivors, facilitating their journey toward posttraumatic growth.

## Data Availability

The data used in the study can be requested from the corresponded author upon reasonable request.
